# microRNA profiles and functions in mosquitoes

**DOI:** 10.1371/journal.pntd.0006463

**Published:** 2018-05-02

**Authors:** Xinyu Feng, Shuisen Zhou, Jingwen Wang, Wei Hu

**Affiliations:** 1 National Institute of Parasitic Diseases, Chinese Center for Disease Control and Prevention, Key Laboratory of Parasite and Vector Biology, National Health and Family Planning Commission, WHO Collaborating Center for Tropical Diseases, National Center for International Research on Tropical Diseases, Shanghai, PR China; 2 Joint Research Laboratory of Genetics and Ecology on Parasites-hosts Interaction, National Institute of Parasitic Diseases—Fudan University, Shanghai, PR China; 3 State Key Laboratory of Genetic Engineering, Ministry of Education Key Laboratory of Contemporary Anthropology, Collaborative Innovation Center for Genetics and Development, School of Life Sciences, Fudan University, Shanghai, PR China; Colorado State University, UNITED STATES

## Abstract

Mosquitoes are incriminated as vectors for many crippling diseases, including malaria, West Nile fever, Dengue fever, and other neglected tropical diseases (NTDs). microRNAs (miRNAs) can interact with multiple target genes to elicit biological functions in the mosquitoes. However, characterization and function of individual miRNAs and their potential targets have not been fully determined to date. We conducted a systematic review of published literature following PRISMA guidelines. We summarize the information about miRNAs in mosquitoes to better understand their metabolism, development, and responses to microorganisms. Depending on the study, we found that miRNAs were dysregulated in a species-, sex-, stage-, and tissue/organ-specific manner. Aberrant miRNA expressions were observed in development, metabolism, host-pathogen interactions, and insecticide resistance. Of note, many miRNAs were down-regulated upon pathogen infection. The experimental studies have expanded the identification of miRNA target from the 3′ untranslated regions (UTRs) of mRNAs of mosquitoes to the 5′ UTRs of mRNAs of the virus. In addition, we discuss current trends in mosquito miRNA research and offer suggestions for future studies.

## Author summary

Millions of human infections are caused by mosquito-borne diseases, so understanding the molecular and genetic mechanisms that determine variability in transmission efficiency and insect susceptibility may assist with novel vector control strategies. Recently, microRNAs (miRNAs) have been studied as post-transcriptional regulators of gene expression in vertebrates, invertebrates, and viruses. Because interactions between vectors and parasites are the keys to malarial transmission dynamics, identifying miRNAs involved in the developmental cycles of the mosquito and interactions between the mosquito and pathogens that allow the survival, proliferation, and disease transmission may help with disease control. In this review, we summarize the research on mosquito miRNAs regarding their expression and function in various physiological and pathological processes, and explore future research in this area.

## Introduction

miRNAs, which are ~22 nt long non-coding RNAs derived from larger hairpin RNA precursors, are involved in the post-transcriptional regulation of target genes in many physiological and pathological processes; therefore, they are of interest as therapeutic targets for treating various diseases [1]. In *Drosophila*, miRNAs control developmental processes, and once they are activated, more than 50 target genes can be regulated temporally and spatially [2]. In mammals, miRNAs may control the activity of ~30% of all protein-coding genes and participate in the regulation of most cellular processes [3].

The mosquito is a vector for numerous crippling diseases, including malaria, West Nile fever, Dengue fever, and other parasitic infections [4]. Studies suggest the worldwide distribution of medically important mosquito species that transmit infectious agents that cause millions of deaths annually. In addition, environmental changes, such as global warming, frequent international travel, and drug resistance, have contributed to keeping mosquito-borne diseases a public health concern [5]. Therefore, we must understand the molecular mechanisms underlying vector biology and host-pathogen interactions to develop novel vector control strategies to reduce disease. Recently, the genome sequences of several important vector mosquitoes have enabled studies of the molecular basis of mosquito feeding, immune function, and development. Studies suggest that miRNA expression in *Anopheles gambiae*, *Anopheles stephensi*, *Aedes aegypti*, *Culex quinquefasciatus*, and *Aedes albopictus* [6–12] have roles in ovary development, blood digestion, and immunity against infections. Furthermore, big data platforms generated from studies of the mosquito genome, transcriptome, and proteome would add in the understanding of vector biology and host-pathogen interactions [13–16].

Studies of miRNAs in mosquito species may provide clues to elucidate their effects on biological functions, invasions of parasites, and induction of immune protection for preventing disease. In this context, we reviewed current literature on mosquito miRNA repertoires and outlined physiological and pathological roles assigned to miRNAs to offer a foundation for future work.

## Methods

A systematic search of the published research for medical subject headings (MeSH) “mosquito” and “microRNA,” “miRNA*,” or “miRNA” was conducted using the electronic online database PubMed. This was supplemented by searches of Google Scholar and Web of Knowledge using the same MeSH terms as well as iterative reviews of reference lists of relevant published papers. After duplicate publications were deleted, all of the records were screened and the abstracts were reviewed if they contained relevant data on mosquito miRNA. Highly relevant papers were selected for full-text reviews. Two reviewers independently extracted and categorized data about the authors, the publication year, country in which the study was performed, and the samples. The characteristics of the study, laboratory methods, miRNAs found, and referred functions were also analyzed. Finally, the reviewers resolved the discrepancies through discussion and consensus.

## Results

**Fig 1:**
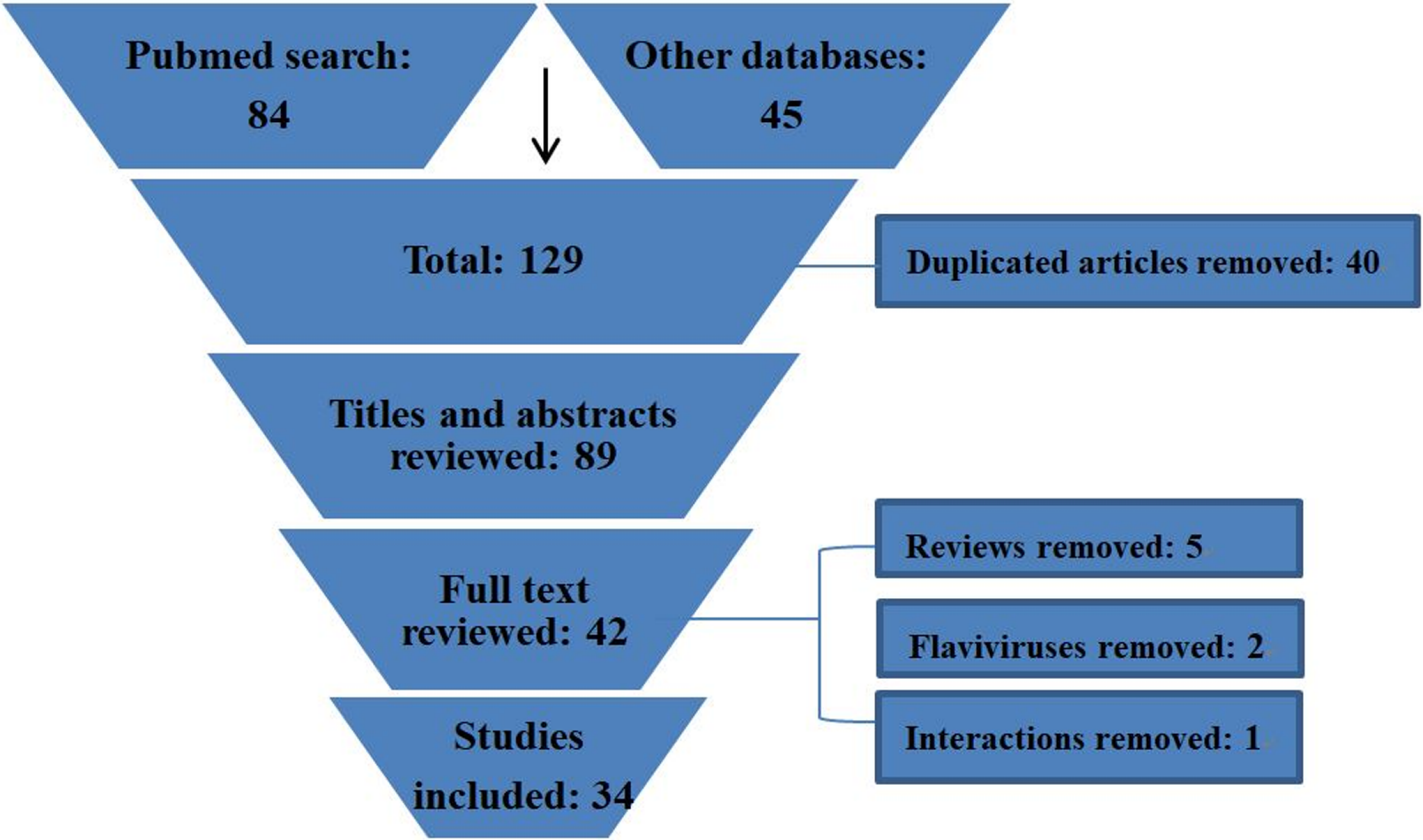
Flow chart diagram of systematic review.

The search process and studies included are depicted in Fig 1. Overall, the search strategy yielded 129 entries. Following the removal of 40 duplicates, 87 titles and abstracts were assessed, and 42 articles appeared to be potentially relevant for inclusion in the review. After eight articles were excluded based on the exclusion criteria, there were 34 articles that fulfilled the eligibility criteria, and these were included in the analysis.

We located 1,540, 1,893 and 383 putative miRNAs from the *Anopheles* 16 genomes project (mostly through computational methods), VectorBase, and miRBase databases, respectively (Table 1). Table 2 depicts 29 experimental studies that mention miRNA profiles in the tissues or organs of many mosquito species. These selected experimental studies were stratified into seven categories according to mosquito species: *Aedes aegypti* (10), *Aedes albopictus* (8), *Anopheles gambiae* (4), *Anopheles stephensi* (4), *Culex quinquefasciatus* (1), *Culex pipiens* (2), and *Anopheles anthropophagus* (1) as shown in Table 2. Overall, over 800 distinct mosquito miRNA sequences have been identified by experimental studies in *Aedes*, *Anopheles*, and *Culex* subgenera (Table 2).

**Table 1:** Mosquito miRNAs deposited in different databases (as of October 12, 2016).

Mosquito species	Number of miRNAs from the *Anopheles* 16 genomes project	Number of miRNAs in VectorBase (strain)	Number of miRNAs records in miRBase
*Aedes aegypti*	-	115 (Liverpool)	101 precursors
			124 mature
*Aedes albopictus*	-	-	-
*Culex quinquefasciatus*	-	92 (Johannesburg)	74 precursors
			93 mature
*Anopheles albimanus*	96	79 (STECLA)	-
*Anopheles arabiensis*	95	97 (Dongola)	-
*Anopheles atroparvus*	96	64 (EBRO)	-
*Anopheles christyi*	93	84 (ACHKN1017)	-
*Anopheles coluzzii*	43	-	-
*Anopheles culicifacies*	71	91 (A-37)	-
*Anopheles darlingi*	55	106 (Coari)	-
*Anopheles dirus*	64	73 (WRAIR2)	-
*Anopheles epiroticus*	85	88 (Epiroticus2)	-
*Anopheles farauti*	108	75 (FAR1)	-
*Anopheles funestus*	76	79 (FUMOZ)	-
*Anopheles gambiae-PEST*	63	116 (PEST)	66 precursors
			65 mature
*Anopheles gambiae-S*	61	-	-
*Anopheles maculatus*	40	60 (maculatus3)	-
*Anopheles melas*	65	95 (CM1001059_A)	-
*Anopheles merus*	118	95 (MAF)	-
*Anopheles minimus*	48	77 (MINIMUS1)	-
*Anopheles quadriannulatus*	70	90 (SANGWE)	-
*Anopheles sinensis*	115	88 (SINENSIS)	-
		76 (China)	
*Anopheles stephensi*	78	77 (SDA-500)	-
		76 (Indian)	

miRNAs annotated in the *Anopheles* 16 genomes project, miRBase, and VectorBase databases.

**Table 2:** Stratification of miRNA studies by the experimental approach based on mosquito species.

Mosquito species	No. of miRNAs*	Related infectious agent/function	References
*Aedes aegypti*	86 known	DENV-2, *Wolbachia*	[9][17–23]
	31 novel	WNV, CHIKV, Blood feeding, Reproduction	[24, 25]
*Aedes albopictus*	103 known	DENV-2, WNV, CHIKV, Blood feeding, Reproduction	[12, 26–28]
	5 novel		[24, 29–31]
*Anopheles anthropophagus*	81 known	Development	[32]
	21 novel		
*Anopheles gambiae*	123 known	*P*. *berghei*, *P*. *falciparum*, Blood feeding, Reproduction	[6, 13, 33, 34]
	58 novel		
*Anopheles stephensi*	111 known	*P*. *vinckei petteri*, Blood feeding, Reproduction	[7, 8, 35, 36]
	17 novel		
*Culex pipiens pallens*	100 known	Pyrethroid resistance	[10, 37]
	42 novel		
*Culex quinquefasciatus*	77	WNV	[30]

*No. of miRNAs means the number of miRNAs; it is the maximum number of miRNAs identified from the studies.

**Fig 2:**
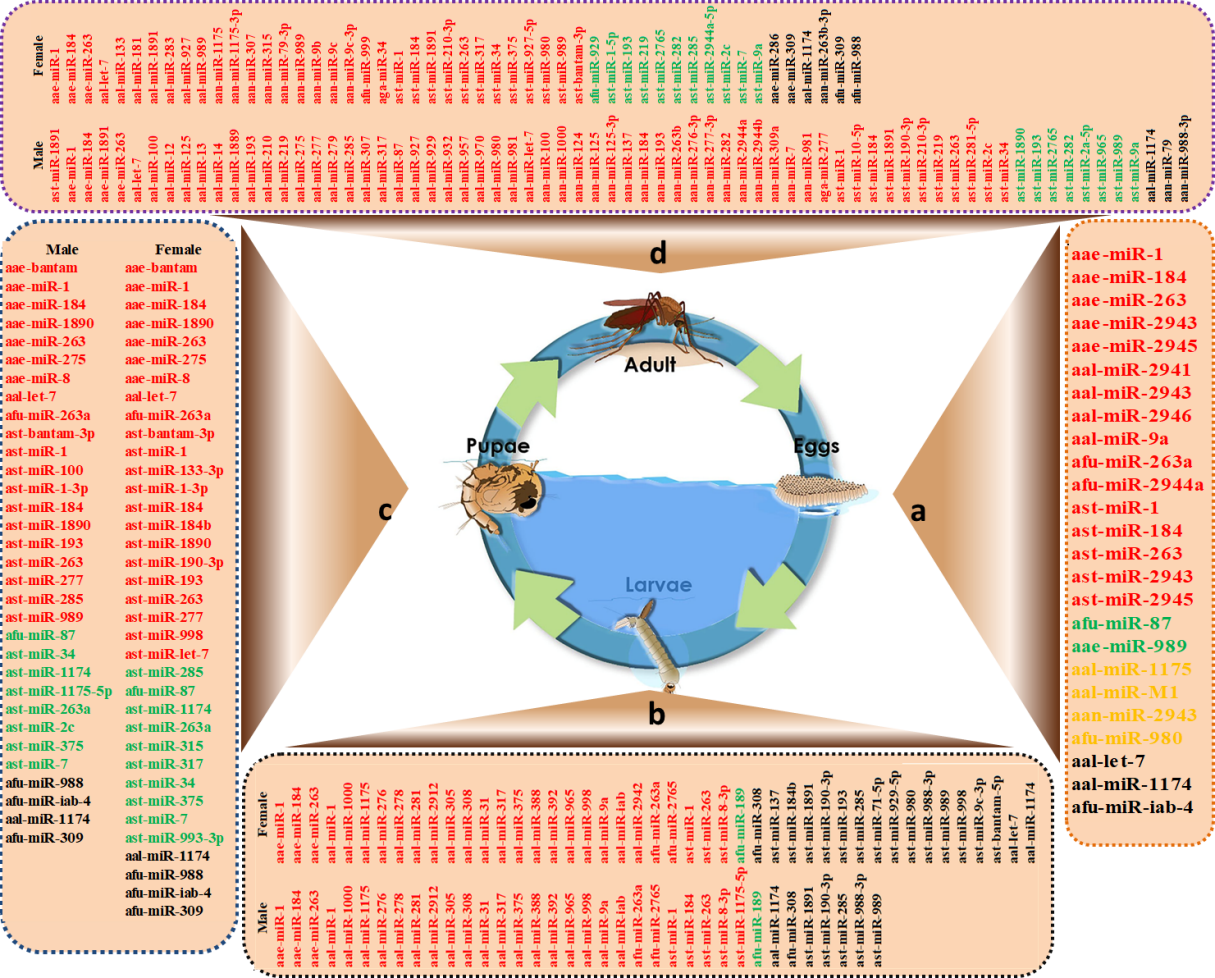
Differentially expressed miRNAs in the mosquito life cycle from egg to adult. a) Differentially expressed miRNAs in eggs; b) Differentially expressed miRNAs in larvae; c) Differentially expressed miRNAs in pupae; and d) Differentially expressed miRNAs in adults. The red color represents highly expressed miRNAs, the green color represents low expressed miRNAs, the yellow color represents exclusively expressed miRNAs, and the black color represents no miRNA expression.

**Fig 3:**
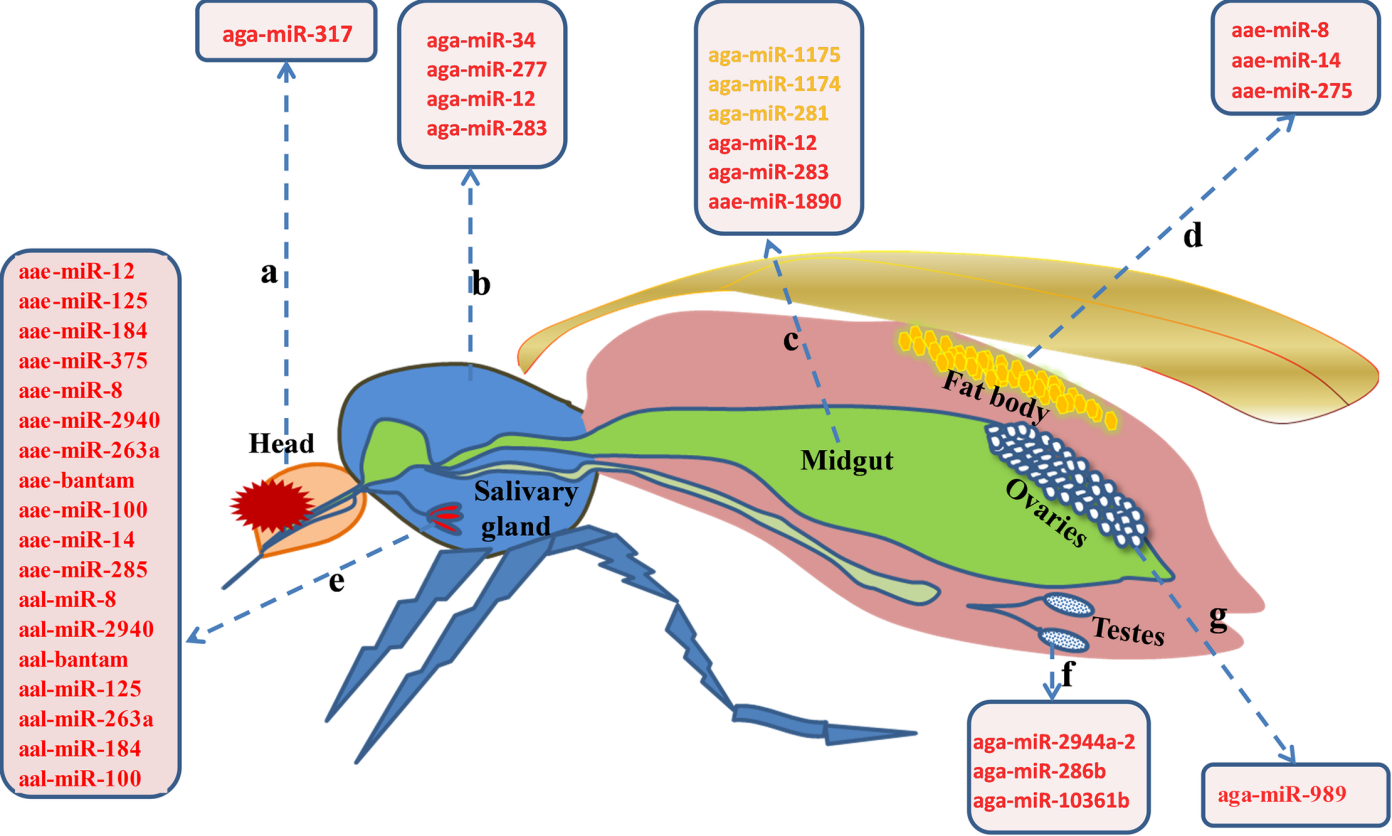
Differentially expressed miRNAs in mosquito tissues or organs. a) Differentially expressed miRNAs in the mosquito head; b) Differentially expressed miRNAs in the mosquito thorax; c) Differentially expressed miRNAs in the mosquito gut; d) Differentially expressed miRNAs in the mosquito fat body; e) Differentially expressed miRNAs in mosquito salivary glands; f) Differentially expressed miRNAs in mosquito testes; and g) Differentially expressed miRNAs in mosquito ovaries. The red color represents highly expressed miRNAs and the yellow color represents exclusively expressed miRNAs.

**Fig 4:**
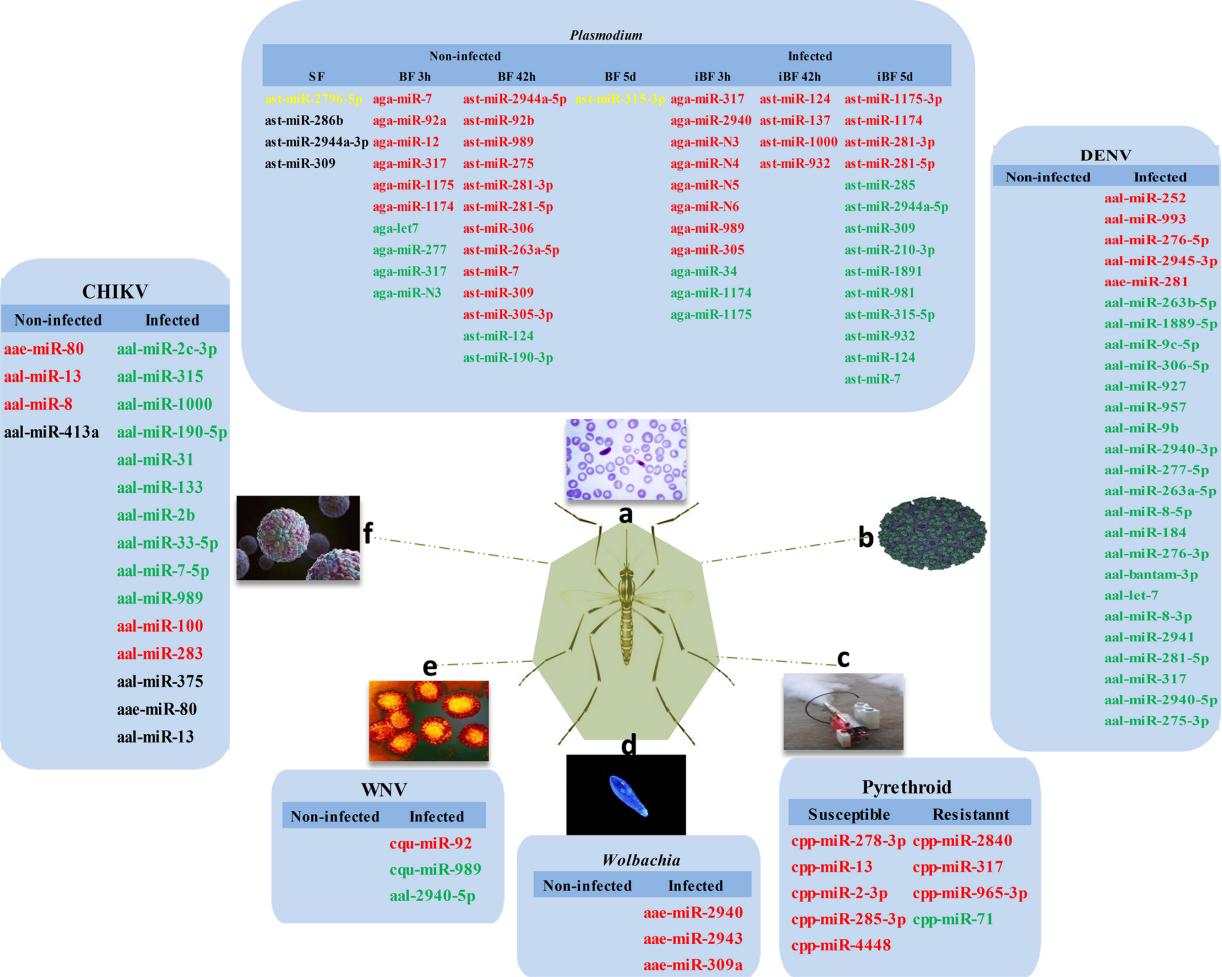
Differentially regulated miRNAs in mosquitoes upon pathogen infection and between pyrethroid-susceptible and -resistant mosquitoes. a) Differentially regulated miRNAs in mosquitoes upon *Plasmodium* infection; b) differentially regulated miRNAs in mosquitoes upon DENV infection; c) differentially regulated miRNAs between pyrethroid-susceptible and -resistant mosquitoes; d) differentially regulated miRNAs upon *Wolbachia* infection; e) differentially regulated miRNAs in mosquitoes upon WNV infection; and f) differentially regulated miRNAs in mosquitoes upon CHIKV infection. The red color represents up-regulated miRNAs, the green color represents down-regulated miRNAs, the yellow color represents exclusively expressed miRNAs, and the black color represents no miRNA expression.

Fig 2 depicts differentially expressed miRNAs that are sex-specific in the mosquito life cycle from egg to adult. Mosquito body elements, such as the head, thorax, gut, and ovary, may have distinct expression profiles and these data appear in Fig 3. miRNAs with roles in interactions between mosquitoes and pathogens appear in Fig 4. The predicted miRNA targets in mosquitoes and the targets of regulated miRNAs identified at this time appear in Table 3.

**Table 3:** miRNAs with validated functions in mosquitoes.

Name	Target	Function	Mosquito species	References
aae-miR-1890	3' UTR of *JHA15* mRNA	Regulates blood digestion	*Ae*. *aegypti*	[17]
aae-miR-8	*Secreted wingless-interacting molecule* (*swim*)	Regulates productive events	*Ae*. *aegypti*	[18]
cpi-miR-278-3p	*CYP6AG11*	Regulates pyrethroid resistance	*Cu*. *Pipiens pallens*	[10]
aal-miR-281	5’-UTR SLA structure of DENV-2 (nt37–nt55)	Enhances DENV-2 viral replication	*Ae*. *albopictus*	[27]
aal-miR-252	*DENV-2 envelope* gene	Regulates gene expression of DENV-2 E protein	*Ae*.*albopictus*	[11]
aae-miR-2940-5p	*Metalloprotease* (*MetP*); *methyltransferase Dnmt2*; arginine methyltransferase	Inhibits WNV replication; *Wolbachia* maintenance; affects arbovirus replication	*Ae*.*aegypti*	[21, 38–40]
aae-miR-1174	*Serine hydroxymethyltransferase* (*SHMT*)	Regulates related gut functions, including sugar absorption, fluid excretion, and blood intake	*Ae*.*aegypti*	[19]
aae-miR-12	*DNA replication Licensing factor* (*MCM6*) and (*monocarboxylate transporter*) *MCT1* genes	Affects *Wolbachia* density in the host cells	*Ae*. *aegypti*	[41]
aae-miR-275	*20-hydroxyecdysone* (20E) and *amino acid/target of rapamycin* (*AA/TOR*)	Involved in blood digestion, fluid excretion, and egg development	*Ae*. *aegypti*	[23]
aae-miR-309	*SIX4*	Controls ovarian development	*Ae*.*aegypti*	[42]
cpi-miR-71	*cytochrome P450 325BG3* (*CYP325BG3*)	Involved in deltamethrin resistance	*Cu*. *pipiens*	[37]
aae-miR-375	*cactus* and *REL1*	Enhances Dengue virus serotype2 (DENV-2) infection	*Ae*. *aegypti*	[22]

## Discussion

We offer a review of miRNAs for mosquito vectors of infectious agents and note that miRNAs were dysregulated in a species-, sex-, stage-, and tissue/organ-specific manner. Of the miRNAs identified, mir-281, mir-184, mir-989, and mir-278 were the most highly expressed miRNAs in the reported studies. Of the miRNAs dysregulated under different physiological conditions and in the presence of infectious agents, many known miRNAs increased after blood feeding. Of note, many miRNAs were down-regulated upon pathogen infection. In addition, 11 evidence-based targets were identified in three mosquito species from 34 studies.

Similar to insects and other animals, miRNAs in mosquitoes are single-stranded 22- to 24 nt non-protein coding RNAs that can inhibit protein translation or promote the degradation of targeted mRNAs by recognizing and binding to the 3´ UTR regions of the target mRNAs. Studies of mosquito miRNA are increasing and in 2005, mosquito miRNA studies began using a genome-wide computational approach to predict miRNAs based on sequence information and the structural characteristics of known miRNAs [43]. Then, *An*. *gambiae* miRNAs were identified by using shot-gun cloning and bioinformatics analysis [6]. Since then, next-generation sequencing (NGS) has helped to update mosquito miRNA profiles and reveal their roles in regulating reproductive processes and pyrethroid resistance, as well as pathogen infection [9–11, 18, 27, 30, 36].

We found 5 studies [14, 43–46] covering 19 *Anopheles* species, and miRNA profiles were identified using computational approaches from sets of primary NGS reads, transcriptome sequences, and Expressed Sequence Tags (ESTs) by combining sequence-alignment-based methods and secondary structural features in homolog searches. A comparative investigation between species identified evolutionarily conserved miRNAs and new miRNAs. Among them, the international “Anopheles Genomes Cluster Consortium” project (a.k.a. “*Anopheles* 16 Genomes Project”) [14] yielded putative miRNAs, as shown in Table 1. In addition, some computational methods based on different algorithms have been applied to predict distinct miRNAs of several mosquito species [44, 45, 47], but comparatively few have been identified by considering the miRNAs of human or mouse (~ 2,000). miRNAs for mosquitoes approximate those found for *D*. *melanogaster*, [48] and some miRNAs are highly evolutionarily conserved.

Table 1 depicts VectorBase and miRBase data. Although disagreements exist between computationally determined miRNAs and those identified by experiments, the total number of miRNAs identified in mosquitoes are consistent. In fact, 20 *Anopheles* mosquito miRNA profiles from the *Anopheles* 16 genomes project are putative miRNA genes derived from a bioinformatics pipeline and some have been annotated as miRNAs in miRBase [48] or other databases [49]. Tables 1 and 2 show overlapping miRNAs identified in *An*. *gambiae* and *An*. *Stephensi*. Currently, computational methods and NGS are used to confirm miRNA; computational methods can overcome limitations of species and expressed miRNAs while NGS has advantages for expanding the repertoire of conserved and species-specific miRNAs in various mosquito species.

### Species-specific miRNAs

Approximately 3,000 mosquito species from 34 genera exist and some are disease vectors. Previous studies revealed genetic determinants that affect the ability of different strains of mosquitoes to transmit pathogens, such as gene profiles [50] and transcriptomes [51]. miRNAs are key to the regulation of gene expression at transcriptional and post-transcriptional events and advances in miRNA have illuminated a role for these small RNAs in development and vector-pathogen interactions.

miRNA types and amounts (overall number of distinct miRNAs) vary across mosquito species (as shown in Table 1). Direct sequencing revealed variations in miRNA profiles among different mosquito species. By analyzing the miRNA profiles of three mosquito species using the miRBse database, we found 52 miRNAs shared among three mosquito lineages (*Aedes aegypti*, *Culex quinquefasciatus*, and *Anopheles gambiae*), and others were specific to certain mosquitoes, for example, aae-miR-2940, aae-miR-2943, and aae-miR-2945 were only observed in *Aedes aegypti*. Of 111 known miRNAs expressed across developmental stages *An*. *stephensi* [7], 103 were identified in in *Ae*. *albopictus* [52]. Hu’s group identified 7 and 19 miRNAs unique to *Ae*. *aegypti* and *An*. *stephensi*, respectively [35]. Interestingly, although *Ae*. *aegypti* and *Ae*. *albopictus* are related, aae-miR-1174 was not found in *Ae*. *albopictus* developmental stage libraries [29].

Mosquitoes appear to have retained highly conserved miRNAs during their evolution. Homologous miRNAs identified in mosquito species indicate evolutionary pressure for miRNA sequence conservation and potentially critical functions of these miRNAs. Among conserved miRNAs, some were shared among many species, such as mir-281, mir-184, mir-989, and mir-278, which are generally expressed in *An*. *gambiae* [13], *Ae*. *Aegypti*, and *Cu*. *quinquefasciatus* [37]. The expression patterns of conserved miR-14, miR−184, miR-210, miR-970, and miR-998 in *Ae*. *aegypti* are similar to the patterns found in *An*. *stephensi* [9]. However, novel miRNAs, usually identified as subsets of differentially expressed miRNAs, had distinct characteristics; for example, aae-mir-2946, which could only be found in *Ae*. *aegypti* [9], and cqu-mir-2951 and cqu-mir-2952, which are only found in *Cu*. *quinquefasciatus* [30]. Sequence analysis of novel miRNAs indicates that they often lacked orthologs found in other mosquito species. Novel miRNAs are potentially restricted to certain species but they are less abundant than known conserved miRNAs [37]. Although *Anopheles*, *Aedes*, and *Culex* genera may have shared a common ancestor approximately million years (MYr) ago [53], each lineage has specific miRNAs, indicating a loss or gain of these miRNAs in species to achieve and control different functions (S1 Table).

### Stage-specific miRNAs

An investigation of stage-specific miRNAs may provide an understanding of mosquito biology and provide mosquito-specific targets for disease control. Significant stage-specific expression was observed for miRNAs in various species (Fig 1). In *Anopheles*, aan-miR-2943 and afu-miR-980 were only expressed in the egg stage in *An*. *anthropophagus* [32] and *An*. *funestus*, [54] respectively. ast-miR-2943 and ast-miR-2945 were highly expressed in *An*. *stephensi* embryos, and ast-miR-1890 had a peak expression in *An*. *stephensi* pupae [35]. Jain and colleagues [7] reported that 36 miRNAs were differentially expressed among various developmental stages of *An*. *stephensi*, including larval male and female, pupal male and female, and adult male and female. Among them, ast-miR-1891, ast-miR-190-3p, ast-miR-285, ast-miR-988-3p, and ast-miR-989 were absent in the larval stage, but ast-miR-8-3p was the most abundant in the male and female larval stages. ast-bantam-3p was the most abundant in the male and female pupal stages of development, and ast-miR-281-5p and ast-miR-bantam-3p were the most abundant in adult males and females, respectively. ast-miR-14 had a relatively strong signal from the late embryonic to adult stages [36]. The consistent expression of ast-miR-14 suggests that it may be essential throughout development, from embryos to aged adults.

Expression analysis of miRNAs revealed distinct patterns from early embryo to adult stages in Aedes. In *Ae*. *albopictus*, aal-mir-M1 was only expressed in embryos, and aal-mir-9a was mainly expressed in embryo and larval stages. aal-let-7 was only expressed in pupal and adult stages, and aal-miR-1175 was widely expressed in all of the life stages, except for embryos [31]. There was aal-miR-286b accumulation in the embryo and aal-miR-2942 was the most expressed in larvae although it was normally expressed at the egg, pupae, and adult stages. aal-miR-1891 was more expressed in adult females than adult males, suggesting a possible regulatory role in blood feeding and egg development [12]. In addition, the increased expression of aal-miR-2941, aal-miR-2943, and aal-miR-2946 occurred in embryos, [29] which is consistent with the results for *Ae*.*aegypti* [9]. In *Ae*. *aegypti*, aae-miR-275 was required for egg maturation, but aae-miR-bantam, aae-miR-275, and aae-miR-8 were highly expressed during the pupal period. However, aae-miR-275 was prominent at the beginning of the pupal stage, and aae-bantam and aae-miR-8 peaked at the mid-pupal stage [23]. The expression of the same miRNAs may differ across stages. Li and colleagues [9] found that aae-miR-989 had 2 read counts in the embryo stage, but 33 read counts in sugar-fed *Ae*.*aegypti* female adults.

Conserved miRNAs are likely to be involved in important functions in mosquito lineages. In *Ae*. *aegypti* and *An*. *stephensi*, miR-2943 and miR-2945 were highly expressed in embryos. Bantam and miR-1890 were highly expressed during the pupal developmental period, and miR-1891 was most abundantly expressed in adult males [36] [35]. Therefore, the expression of some stage-specific miRNAs may be conserved in most lineages and stage-specific miRNAs may be involved in the regulation of growth, differentiation, and reproduction during a specific developmental stage.

### Sex-specific miRNAs

Understanding how sex-specific miRNA expression occurs in mosquitoes (Fig 2) has great significance towards its role in blood feeding and disease transmission. For instance, 29 miRNAs (based on read count of miRNAs) had sex-biased expression in *An*. *anthropophagus* [32]; of these, 9 miRNAs were up-regulated in females and 20 miRNAs had decreased or no expression. Among them, aan-miR-989 was highly expressed in female mosquitoes, but not in males—similar to patterns in *An*. *gambiae* [6]. In other studies, miR-989 was up-regulated in adult female *An*. *stephensi* [7] and *Ae*. *aegypti* [36] compared to adult males, suggesting functional conservation among mosquitoes. In *An*. *gambiae*, the expression of aga-miR-34 was more pronounced in the midguts of females, while aga-miR-277 was highly expressed in the midguts of males [6]. miR-1891 was most abundantly expressed in *Ae*. *aegypti* and *An*. *stephensi* adult males [35]. In addition, Northern blot and sequencing counts indicated that the expression of miR-184 and miR-1000 in male adults was higher than in female adults in *Ae*. *Aegypti* [9] and *An*. *anthropophagus* [32].

Moreover, sex-specific miRNA expression diverged during larval, pupal, and adult mosquito stages [7]. Fewer miRNA differences were identified during *An*. *stephensi* immature stages, and two miRNAs (ast-miR-184b and ast-miR-1175-5p) were up-regulated in male larvae; one miRNA (ast-miR-285) was down-regulated in the female pupal stage. Maximal differences in miRNA expression between sexes were observed during the adult stages, except for ast-miR-989, and several miRNAs were down-regulated in female *An*. *stephensi*, including miR-7, which was also reported in *An*. *anthropophagus* [32]. The expression of miR-989 was restricted to adult females and predominantly in the ovaries of *Anopheles* and *Aedes*. Mead’s group observed reduced miR-989 in post-blood-meal (PBM) females (72 h) [36]. To investigate the role of sex-specific miRNAs in mosquito reproduction, Jain’s group [7] injected miR-989-specific antagomirs in female mosquitoes and their expression affected multiple functions in ovaries after blood-feeding. Thus, miR-989 may be associated with female reproduction, and its function may be conserved among divergent mosquitoes.

### Tissue/Organ-specific miRNAs

Different body parts of the mosquito, such as the head, thorax, gut, and ovary, have distinct expression profiles (Fig 3). aga-miR-317 was more expressed in the head compared to the thorax, leftover (carcass), and midgut [6]. The preferential expression of aga-miR-34, aga-miR-277, aga-miR-12, and aga-miR-283 occurred in the thorax of both males and females, and twice as much in heads.

Midgut-specific miRNAs have been identified in *An*. *gambiae*, *Ae*. *Albopictus*, and other mosquito species. For instance, aga-miR-12 and aga-miR-283 were predominantly expressed in the midgut. aga-miR-1175, aga-miR-1174, and aga-miR-281 were expressed only in the midgut. In addition, the miR-1174/miR-1175 miRNA cluster was highly expressed in *An*. *gambiae* gut PBM [6]. In *Ae*. *albopictus*, the midgut-specific aal-miR-281 was the most abundant miRNA. A high expression of aae-miR-1890 was observed in the midgut of female *Ae*. *aegypti*, and mature aae-miR-1890 peaked at 24 h PBM and declined sharply by 36 h PBM in the female mosquito midgut [17]. In addition, miR-281, miR-1174, and miR-1175 were also only found to be expressed in the midgut of adults in *Ae*. *aegypti*, *Ae*. *albopictus*, and *Cu*. *quinquefasciatus* [9] [30].

In *An*. *stephensi* and *Ae*. *aegypti*, the expression of miR-989 was predominantly in the ovaries [36]. aae-miR-8, aae-miR14, and aae-miR-275 were highly expressed in the vitellogenic fat body [23], and aae-miR-8 was substantially increased PBM in female *Ae*. *aegypti* fat bodies [28] [23]. In addition, 41 miRNAs were differentially expressed in the testes and pre-vitellogenic ovaries. Among them, aga-mir-2944a-2 and aga-mir-286b were up-regulated in the testes and during oogenesis, suggesting a role in gametogenesis [34]. Then, 103 extracellular miRNAs were identified from *Ae*.*aegypti* and *Ae*.*albopictus* saliva; of these, 31 miRNAs were previously unidentified and designated as novel. aae-mir-281-2-5p, aae-mir-281, aae-mir-2940, aae-mir-bantam, aae-mir-125, and aae-mir-263a were highly expressed in uninfected and infected *Ae*. *aegypti* saliva, while aal-mir-8 and aal-mir-125 were equally expressed in uninfected and infected *Ae*. *albopictus* saliva [24]. Therefore, different tissues/organs possess different miRNA expression profiles, and tissue/organ-specific miRNAs may be of more value than some ubiquitously-expressed miRNAs in investigating and explaining specific physiological functions, or as specific indicators to distinguish infections.

### miRNA functions in mosquitoes

The role of miRNAs in the post-transcriptional regulation of gene expression has been recognized to contribute to physiological and immune pathways that affect development, metabolism, host-pathogen interactions, and insecticide resistance.

### Development and metabolism

The stage-specific expression of miRNAs in the four developmental stages (eggs, larvae, pupae, and adults) has been confirmed using high-throughput sequencing followed by Northern blot analysis and quantitative polymerase chain reaction (PCR) [7, 12, 37]. To understand the role of regulated miRNAs in mosquito development, the knock-in and knock-down of specifically and temporally expressed miRNAs were conducted in *Ae*. *albopictus* by microinjection. The knock-down of aal-miR-286b and aal-miR-2942 decreased the hatching of embryos and eclosion rate of larvae, respectively, when compared with the knock-in groups. Reduced longevity and fecundity (aal-miR-1891) in adults was observed in the miR-1891 knock-down groups compared to the knock-in and control groups [12]. Female mosquitoes require sugar for energy metabolism and a blood meal for egg development, and recent studies have indicated that blood feeding leads to the differential expression of many genes, proteins, and miRNAs [55–57]. miRNA abundance differs under sugar-fed and blood-fed conditions, and ast-miR-2796-5p was observed exclusively in sugar feeding *An*. *stephensi* with extremely low read counts [8]. aae-miR-375 was only found in blood feeding *Ae*. *aegypti* mosquitoes [22]. Most miRNAs (107) were found in a blood-fed library of *An*. *stephensi* compared with sugar-fed and *Plasmodium*-infected libraries. ast-miR-286b, ast-miR-2944a-3p, and ast-miR-309 were significantly expressed in blood-feeding (BF) 42 h with no reads present in sugar feeding, indicating that the expression of these miRNAs may be induced by a blood meal [8]. Expressions of 4 miRNAs (aga-miR-7, aga-miR-92a, aga-miR-317, and aga-miR-N3) were significantly changed in blood-fed *An*. *gambiae* [13]. Expression changes occurred in aga-miR-34 and aga-miR-989 in leftovers and midguts [6].

Moreover, variations in miRNA expression are temporally regulated. aae-miR-275, which is required for blood digestion in *Ae*. *aegypt*, was elevated 7.2-fold from 0 to 12 h PBM [23]. The depletion of aae-miR-275 in *Ae*. *aegypti* females by injection of its specific antagomir led to severe defects in blood digestion, fluid excretion, and egg development. aae-miR-1890 is induced after blood feeding and peaks at 24 PMB, and systemic depletion of aae-miR-1890 resulted in decreased egg development and deposition, suggesting that miR-1890 may be key to mosquito blood digestion [17]. In contrast with up-regulated miRNAs after blood feeding, some miRNAs were down-regulated. For example, reduced ast-miR-989 was observed 72 h after a blood meal [36]. aga-let7 was decreased in the midguts and other parts/leftovers [6], but most miRNAs were increased after blood feeding [9].

### *Plasmodium* infection

The malarial vector *Anopheles* initiates strong immune responses by inducing the expression of key anti-*Plasmodium* effectors upon the invasion of *Plasmodium* parasites, which are largely regulated by 3 immune signaling pathways, namely, the Toll, Jak/Stat, and immune deficiency (IMD) pathways [58, 59]. miRNAs may fine-tune immune responses and other physiological processes. The expression of aga-miR-34, aga-miR-1174, and aga-miR-1175 decreased in the midgut after *P*. *falciparum* infection, while aga-miR-989 and aga-miR-305 were elevated in infected midguts. A functional study showed that aga-miR-305 increased susceptibility to *P*. *falciparum* infection and proliferated midgut microbiota [33]. Infection of *An*. *stephensi* and *An*. *gambiae* with the rodent malarial parasite *P*. *vinckei petteri* and *P*. *berghei* caused the differential expression of multiple miRNAs [8, 13]. For instance, 6 miRNAs were significantly up-regulated after *P*. *berghei* infection[13]; of these, aga-miR-317 and aga-miR-2940 were more than 5- and 3-fold unregulated. Then, 4 miRNAs were markedly up-regulated in infectious blood feeding 42 h (ast-miR-124, ast-miR-137, ast-miR-1000, and ast-miR-932) and 5 d (ast-miR-1175-3p, ast-miR-1174, ast-miR-281-3p, and ast-miR-281-5p) of infectious blood-feeding. Meanwhile, 10 miRNAs (ast-miR-285, ast-miR-2944a-5p, ast-miR-309, ast-miR-210-3p, ast-miR-1891, ast-miR-981, ast-miR-315-5p, ast-miR-932, ast-miR-124, and ast-miR-7) were significantly down-regulated in the infectious blood feeding 5 d group compared with the 42 h group after *P*. *vinckei petteri* infection [8]. In addition, Dicer1, Dicer2, Drosha, and Ago1 are involved in miRNA biogenesis and increased polysome loading after infection in mosquitoes. The knock-down of Dicer1 and Ago1 changed mosquito susceptibility to the *Plasmodium* parasite [6] [60]. Thus, mosquito miRNAs may participate in reactions against *Plasmodium* invasion.

### WNV and DENV infection

Flavivirus genus viruses are spread by mosquitoes and cause diseases, including Dengue and West Nile fever. To determine whether flavivirus infection could alter miRNA expression, Skalsky’s group infected female *Cu*. *quinquefasciatus* mosquitoes with WNV (West Nile virus), and cuq-miR-92 and cuq-miR-989 had altered expressions [30]. Slonchak’s group found aae-miR-2940 was selectively down-regulated in *Aedes albopictus* cells in response to WNV infection to restrict viral replication [38]. Campbell and co-workers observed that the expressions of 35 mosquito miRNAs were modulated upon DENV (Dengue virus) infection in *Aedes aegypti*s, [20] and Liu’s group noted that 66 miRNAs of *Ae*. *albopictus* were differentially expressed after DENV-2 infection [52]. Therefore, aal-miR-34-5p and aal-miR-87 may contribute to anti-pathogen and immune responses during DENV-2 infection [52]. aae-miR-375 is the key to DENV replication, which may enhance DENV-2 infection in an *Ae*.*aegypti* cell line [22]. aae-miR-252 was induced more than three-fold after DENV-2 infection in an *Ae*. *albopictu*s C6/36 cell line, which inhibited DENV replication by suppressing the expression of the DENV envelope protein [11]. aal-miR-281, an abundant midgut-specific miRNA, facilitates DENV-2 replication in *Ae*. *albopictus* [27].

### CHIKV infection

Chikungunya virus (CHIKV) is a alphavirus transmitted predominantly by *Aedes aegypti* and *Aedes albopticus*, and it causes severe symptoms, including the risk of death [24]. Shrinet’s group evaluated the role of host miRNAs upon CHIKV infection in *Ae*. *albopictus* and they observed an altered expression of 8 miRNAs [61]. Maharaj and co-workers reported 59 and 30 miRNAs upregulated in *Ae*.*aegypti* and *Ae*. *albopictus* CHIKV-infected saliva, respectively, indicating the importance of saliva miRNAs in regulating CHIKV infection in mammals [24].

### *Wolbachia* infection

*Wolbachia* are widespread in invertebrates and can manipulate reproduction, reduce the host life span, and inhibit pathogen infections, such as DENV, filarial nematodes, and malarial parasites [21, 62, 63]. In 2011, a microarray analysis of miRNAs revealed that ~13 miRNAs were differentially expressed in *Wolbachia*-infected female *Ae*. *aegypti* mosquitoes [39]. Also, aae-miR-12 was differentially expressed in *Ae*. *aegypti* infected with *Wolbachia*. Then, Osei-Amo found that the inhibition of aae-miR-12 reduced *Wolbachia* density in *Wolbachia*-infected Aag2 mosquito cell lines [41]. Decreased aae-miR-2940 and aae-miR-184 was observed in *AGO2*-silenced and *Wolbachia*-infected cells [22]. Then, aae-miR-2940 was induced and exclusively found in *Wolbachia*-infected mosquitoes [39]. *Wolbachia* uses host aae-miR-2940 to regulate a methyltransferase gene to block DENV replication [40]. aae-miR-989, aae-miR-306-5p, and aae-miR-1889 were down-regulated in *Wolbachia*-infected *Ae*. *aegypti*, while aae-miR-2765 and aae-bantam-5 were up-regulated [25]. Therefore, *Wolbachia* influences miRNA expression and alters natural miRNA profiles in the mosquito.

### Pyrethroid resistance

Pyrethroid resistance due to excessive and improper usage of pyrethroids is an impediment to combating mosquito-borne diseases. To validate whether miRNAs have a role correlated with insecticide resistance, Lei’s group measured miRNA expression in pyrethroid-resistant and susceptible strains of lab populations and confirmed the dysregulated miRNAs. Of these, miR-278-3p was up-regulated in the susceptible *Culex pipiens pallens* strain [10]. In another study, cpi-miR-71 was significantly down-regulated in female adults from a deltamethrin-resistant strain, indicating that cpi-miR-71 may play a contributing role in deltamethrin resistance [37]. Then, the overexpression of cpi-miR-71 in female mosquitoes had reduced resistance to deltamethrin. Differentially expressed miRNAs in these studies provide a basis for the investigation of pyrethroid resistance in the future.

### miRNA target in mosquitoes

To understand the role of regulated miRNAs in development, sugar feeding, blood feeding, and pathogen invasion, we must identify relevant targets. Studies show that bioinformatic analysis and *in vivo* assays can be used to identify the targets of regulated miRNAs. Targets were predicted by identifying miRNA seed-binding sites on the 3' UTR of genes using RNAhybrid [64], miRanda [65], TargetScan [66], PicTar [67], and other in-house pipelines. Dual-luciferase reporter assays to assess target identification were used, and degradome sequencing has recently been used to identify cleaved targets of regulated miRNAs by sequencing degraded mRNA [7]. This may allow researchers to overcome the limits of bioinformatic predictions and locate target genes for miRNAs.

Studies to predict miRNA targets in mosquitoes and targets of regulated miRNAs have been identified (Table 3). Ovary-specific aae-miR-309 was found to target *SIX4* and contribute to *Ae*. *aegypti* mosquito reproduction [42]. The ortholog of *SIX4* in *D*. *melanogaster* is required for gonadogenesis [42]. Blood-feeding in mosquitoes is a major metabolic challenge, and aae-miR-1890 was shown to bind the 3' UTR of *JHA15* mRNA (with presumed role in blood digestion) and control *JHA15* mRNA stability in a stage- and tissue-specific manner to regulate blood digestion [17]. In addition, secreted wingless-interacting molecule (*swim*), an important gene that could interrupt Wg signaling activity in *Drosophila*, was regulated in female mosquito fat body [18]. Pathogenic agents can alter host-derived miRNAs, which then modulate the host gene expression to cause translational inhibition and mRNA decay. *Cactus* and *REL1* genes were targets of blood-induced aae-miR-375, and the injection of an miRNA mimic into mosquitoes led to fold-changes in immune gene transcripts, suggesting that aae-miR-375 enhanced DENV-2 infection [22]. Furthermore, three targets of aae-miR-2940-5p have been validated. Metalloprotease ftsh (*MetP*) was found to be the first target of miRNA and it was important for the replication/maintenance of *Wolbachia* [39]. The second target of aae-miR-2940 was DNA methyltransferase (*Dnmt2*). The overexpression of *Dnmt2* increased DENV-2 replication and reduced *Wolbachia* density [21]. In addition, arginine methyltransferase 3 was found to be a target of aae-miR-2940 [40], which was positively regulated and beneficial for *Wolbachia* replication. Other miRNAs significantly regulated during development or in the presence of pathogens have not been explored. More work is needed to identify their potential roles in metabolic processes, phagocytosis, and immune defense. In addition, miRNAs have been predicted to have multiple gene targets, suggesting the importance of these molecules in regulatory networks.

A comparative analysis of miRNA profiles of different mosquito species revealed that nearly half of known miRNAs are conserved. Conserved miR-184 and miR-998 were identified in *An*. *gambiae*, *Ae*. *aegypti*, *An*. *stephensi*, *Ae*. *albopictus*, and other mosquitoes [9] [24], which indicates evolutionary pressure for miRNA conservation and potentially critical functions of these miRNAs in various species [7]. Species-specific miRNAs are thought to be novel and potentially specific to mosquitoes in low read counts, indicating a loss/gain or rapid change of miRNAs during evolution to achieve and control species-specific functions. However, some highly conserved miRNAs, such as miR-282 and miR-927, found in *Ae*. *aegypti* and *An*. *gambiae* were not confirmed in *Cu*. *quinquefasciatus* [29], indicating that known and novel miRNAs can exhibit species-specific patterns. Species-specific miRNAs may contribute to the susceptibility of different mosquitoes to unique pathogens and mosquito-specific targets for disease control and prevention.

Many miRNAs have spatio-temporal patterns of expression essential for regulating complex physiological activity of mosquitoes. Research has shown a significant reduction in miR-989 72 h after a blood meal in *An*. *stephensi* and *Ae*. *aegypti*, predominantly in adult female ovaries [36]. Some regulated miRNAs were differentially expressed during larval to pupal stages and during pupal to adult metamorphosis [7]. Recent research has indicated possible regulatory effects of aae-miR-8 in reproduction as it is highly expressed in female mosquito fat body PBM [23]. Of note, certain miRNAs (aga-miR-996, aga-miR-279, aga-miR-306, aga-miR-79, aga-miR-9b, and aga-miR-275) were expressed evenly and ubiquitously throughout the *An*. *gambiae* body [6], and most highly expressed miRNAs (miR-1, miR-184, and miR-263) were expressed in most developmental stages in many mosquito species [35]. For example, the consistent expression of ast-miR-14 from the late embryonic to the adult stage indicates that it likely plays an important role across all life stages [36].

Several miRNAs are sexually dimorphic, such as miR-989 with an expression that is restricted to adult *An*. *stephensi* and *Ae*. *aegypti* females[36]. In addition, aga-miR-277 was highly expressed in *An*. *gambiae* [6], and miR-1891 was abundantly expressed in *Ae*. *aegypti* and *An*. *stephensi* adult males [35]. These differences may be a result of unique reproduction strategies and disease transmission. Moreover, several sex-specific miRNAs were observed in pupal, larva, and adult mosquitoes, but embryogenesis has not been investigated and how these are regulated in a sex-specific manner is unclear.

Early studies have identified extracellular miRNAs in saliva and serum in humans and mammals, and these have roles in intercellular communication, coinciding with the transfer of functional and intact proteins, lipids, and nucleic acids between cells. Recent studies identified extracellular miRNAs as being dysregulated in mosquitoes. Maharaj’s group [24] isolated saliva containing extracellular miRNAs from mosquito salivary glands and these were key to pathogen transmission from mosquito to vertebrates. They found 103 mature miRNAs in *Ae*. *aegypti* and *Ae*. *albopictus saliva*. Subsequent experiments confirmed that saliva miRNAs can regulate CHIKV infection. Therefore, extracellular miRNAs may have concomitant changes with intracellular miRNAs and synergistically modulate viral replication.

Aberrant miRNA expression in mammals may be used as a biomarker for disease and some miRNAs can influence the onset and courses of cancer [68] and vascular/heart diseases [69] [70]. miR-21 was misexpressed in diseased hearts [70], and Let7-f, miR-27b, and mir-130a had a proangiogenic role [69]. Studies have shown that infection can alter the expression of mosquito miRNAs. In *Ae*. *aegypti* and *Ae*. *albopictus*, aberrantly expressed miRNA profiles were noted after CHIKV, WNV, and *Wolbachia* infections [23–25]. In *An*. *gambiae* and *An*. *stephensi*, *Plasmodium* infection changed miRNAs expression [11]. While drafting this paper, Saldana’s group reported that the Zika virus modulated 17 host miRNAs in *Ae*. *aegypti* mosquitoes at all post-infection points [71]. Also, many miRNAs are reported to be down-regulated by *Plasmodium*, Dengue, and CHIKV, although responses differed from infections. These data will increase our understanding of pathogen-vector interactions and provide potential avenues to investigate and develop miRNA-based strategies. In particular, Tsetsarkin’s group [72] developed an miRNA-targeted approach by introducing mosquito-specific mir-184 and mir-275 miRNAs to selectively restrict the replication of Dengue type 4 virus (DEN4) in *Ae*. *albopictus* and *Ae*. *aegypti*, which may mitigate the risk of introduction and dissemination.

miRNA profiles have been identified using cloning, microarray or direct sequencing, and influencers of miRNA expression have been reported. However, there is limited research about miRNA target analysis and the gene regulatory networks of miRNA families. There are only a few existing studies regarding specific functions, which limits our understanding of miRNA repertoires and functions in the mosquito. Attention should focus on discovering veiled miRNAs in vector mosquitoes, clarifying the occurrence and spatial-temporal regulation of specific miRNAs, especially miRNA signatures rather than a single miRNA alone. This would allow setting of criteria for target prediction using machine-learning algorithms and exploration of miRNA:mRNA networks in post-transcriptional level. These ideas may spur research to allow miRNAs to be used as vector control tools.

## Supporting information

S1 Checklist PRISMA checklist. (DOC)

S2 Checklist PRISMA flow diagram. (DOC)

S1 Table miRNAs of five main vectors. (XLSX)
