# Supplementary material for: microRNA profiles and functions in mosquitoes
Source: PLoS Negl Trop Dis. 2018 May 2;12(5):e0006463. doi: 10.1371/journal.pntd.0006463 (PMC5951587; doi:10.1371/journal.pntd.0006463)
Supplement: S2 Checklist — (DOC) [file pntd.0006463.s002.doc]

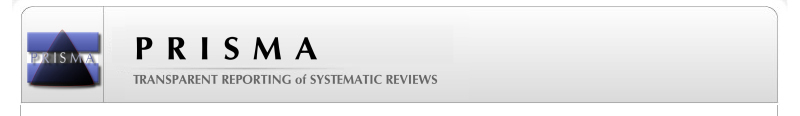
**PRISMA 2009 Flow Diagram**

**Screening**

**Included**

**Eligibility**

**Identification**

Records identified through database searching
(n = 84 )

Additional records identified through other sources
(n = 45 )

Records after duplicates removed
(n = 89 )

Records screened
(n = 89 )

Records excluded
(n = 47 )

Full-text articles assessed for eligibility
(n = 42 )

Full-text articles excluded, with reasons
(n = 8 )

Studies included in qualitative synthesis
(n = 34 )

Studies included in quantitative synthesis (meta-analysis)
(n = 29 )
